# Knowledge-guided multi-scale independent component analysis for biomarker identification

**DOI:** 10.1186/1471-2105-9-416

**Published:** 2008-10-06

**Authors:** Li Chen, Jianhua Xuan, Chen Wang, Ie-Ming Shih, Yue Wang, Zhen Zhang, Eric Hoffman, Robert Clarke

**Affiliations:** 1 Department of Electrical and Computer Engineering, Virginia Polytechnic Institute and State University, Arlington, VA, USA; 2 Departments of Pathology, Gynecology and Oncology, The Johns Hopkins University School of Medicine, Baltimore, MD, USA; 3 Research Center for Genetic Medicine, Children's National Medical Center, Washington, DC, USA; 4 Departments of Oncology and Physiology & Biophysics, Georgetown University School of Medicine, Washington, DC, USA

## Abstract

**Background:**

Many statistical methods have been proposed to identify disease biomarkers from gene expression profiles. However, from gene expression profile data alone, statistical methods often fail to identify biologically meaningful biomarkers related to a specific disease under study. In this paper, we develop a novel strategy, namely knowledge-guided multi-scale independent component analysis (ICA), to first infer regulatory signals and then identify biologically relevant biomarkers from microarray data.

**Results:**

Since gene expression levels reflect the joint effect of several underlying biological functions, disease-specific biomarkers may be involved in several distinct biological functions. To identify disease-specific biomarkers that provide unique mechanistic insights, a meta-data "knowledge gene pool" (KGP) is first constructed from multiple data sources to provide important information on the likely functions (such as gene ontology information) and regulatory events (such as promoter responsive elements) associated with potential genes of interest. The gene expression and biological meta data associated with the members of the KGP can then be used to guide subsequent analysis. ICA is then applied to multi-scale gene clusters to reveal regulatory modes reflecting the underlying biological mechanisms. Finally disease-specific biomarkers are extracted by their weighted connectivity scores associated with the extracted regulatory modes. A statistical significance test is used to evaluate the significance of transcription factor enrichment for the extracted gene set based on motif information. We applied the proposed method to yeast cell cycle microarray data and Rsf-1-induced ovarian cancer microarray data. The results show that our knowledge-guided ICA approach can extract biologically meaningful regulatory modes and outperform several baseline methods for biomarker identification.

**Conclusion:**

We have proposed a novel method, namely knowledge-guided multi-scale ICA, to identify disease-specific biomarkers. The goal is to infer knowledge-relevant regulatory signals and then identify corresponding biomarkers through a multi-scale strategy. The approach has been successfully applied to two expression profiling experiments to demonstrate its improved performance in extracting biologically meaningful and disease-related biomarkers. More importantly, the proposed approach shows promising results to infer novel biomarkers for ovarian cancer and extend current knowledge.

## Background

Under their broadest definition, biomarkers include any biological or chemical indicator of a specific underlying process. In genetics, biomarkers are defined as a set of genes that are associated with a disease or are associated with the susceptibility to develop a specific disease. Microarray technology makes it possible to measure simultaneously the expression levels of thousands of genes, and identifying meaningful and useful biomarkers from these large data sets is a common goal. Specifically, investigators attempt to detect genes differentially expressed across different types of tissue samples or the samples obtained under different experimental conditions. Traditional biomarker identification methods have mainly been applied to statistical analysis of microarray data alone; T-test [1] and significance analysis of microarray (SAM) [2] are frequently used to detect differentially expressed genes between two phenotypes. Several new statistical methods have been developed to analyze time-course microarray data. Storey *et al*. proposed an algorithm (EDGE) to fit the time-course microarray data with natural cubic splines, followed by a goodness-of-fit test to detect differentially expressed genes [3]. Conesa *et al*. also proposed a two-step regression approach to sequentially identify differentially expressed genes from time-course microarray data under different conditions [4]. However, these and many related approaches do not incorporate knowledge of gene function, with respect to the phenotypes of interest, into their statistical models.

Ideally, biomarkers should not only exhibit differential gene expressions between normal and disease samples, but more importantly, they should also reflect their biological role in the disease phenotype. Most significance analysis methods applied to population (static) or time-course microarray data have the limitation that genes are analyzed independently and the interactions among them are ignored. Clustering methods, such as k-means clustering [5] and self-organizing maps (SOMs) [6], were introduced to group the genes with similar expression patterns. A shortcoming of the clustering methods is that they do not allow genes to be shared by multiple clusters. However, a single gene can be involved in multiple distinct biological processes [7]. One solution to this problem is to first infer gene regulatory networks [8-12] that appear to control or regulate phenotypically relevant biological functions, and then to extract the most biologically and statistically relevant biomarkers.

The application of Independent Component Analysis (ICA) to microarray data has shown some utility in regulatory network inference [10,13]. ICA is a statistically-principled linear decomposition method that models the observations as a linear combination of some latent (or hidden) variables [14]. From the perspective of a gene regulatory mechanism, any gene expression value can be regarded as a combinational effect of some regulatory inputs such as transcription factors, cellular functions, or responses to experiment conditions [10,12]. As demonstrated in our previous work [15,16] along with that of others [10,12], novel applications of ICA to high-throughput data from microarray technology can help reveal dominant regulatory mechanisms.

It is not a trivial task to link the estimated latent variables from ICA to real biological functions. To identify biologically relevant biomarkers for a specific disease, the incorporation of prior knowledge is of great importance to improving the accuracy of computational methods [17]. However, complete prior knowledge is often difficult to obtain. Some prior knowledge, such as regulatory motif information (promoter responsive element sequence) is available and can be incorporated into microarray data analysis to assist in regulatory module identification [18,19]. Recently, we have developed a new approach called motif-directed network component analysis (mNCA) to infer transcription regulatory activities (TFAs). This approach incorporates a stability analysis procedure to overcome the problem of many false positives in motif information [20]. Since we can only use known motifs, a clear limitation of the mNCA method is that we cannot infer any new potential regulatory biomarkers beyond prior knowledge from the model.

In this paper, we propose a novel method, namely knowledge-guided multi-scale ICA, to identify disease-specific biomarkers beyond partial prior knowledge. We propose that a latent variable estimated by ICA from the entire gene expression population represents the joint effect of several biological functions. Disease-specific biomarkers could be involved in several different biological functions by the ICA latent variables or linear regulatory modes. Therefore, we first cluster the whole gene population into multiple sub-populations in which only a few biological processes are involved. We then uncover the knowledge-relevant regulatory modes in each subpopulation based on the partial prior knowledge. Finally, disease-specific biomarkers are extracted according to the strength of their association with the extracted regulatory modes. A statistical test is applied to evaluate the significant enrichment of transcription factors for the extracted biomarkers based on motif information.

For algorithm validation, we applied our approach to two time-course microarray data sets to demonstrate its improved performance. The first data set is a yeast cell cycle microarray data set with 104 well known cell cycle-related genes; the second is a remodeling and spacing factor 1 (Rsf-1) induced microarray data set from a profiling study of ovarian cancer. The experimental results show that our approach can identify biologically meaningful disease-specific biomarkers related to ovarian cancer, as compared to other gene selection methods with or without prior knowledge.

## Methods

**Figure 1:**
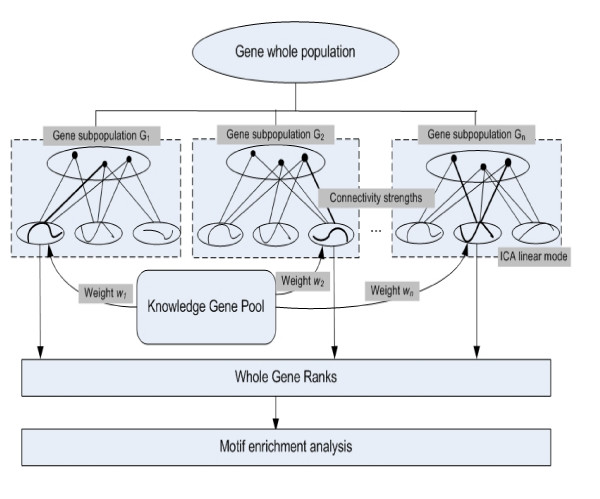
Flow chart of the proposed method – knowledge-guided multi-scale independent component analysis (ICA) – for biomarker identification.

If we apply ICA directly onto an entire gene expression population, the extracted regulatory modes will reflect the joint effect of several biological functions, some of which are related to the disease under study and some are not. To overcome this problem, we developed a divide-and-conquer strategy. We applied a knowledge-guided multi-scale ICA approach to extract disease-related regulatory modes reliably, and then we identify the biomarkers associated with the modes. The overall scheme is illustrated in Fig. 1. Firstly, a knowledge gene pool (KGP) is constructed by collecting the genes that are known to be relevant to the specific disease from available databases and literatures. Secondly, the entire gene population is divided into sub-populations by a clustering method applied to the microarray data and, to identify regulatory modes, ICA is applied to each sub-population. The most relevant linear regulatory mode in each cluster is extracted using the gene metadata in the KGP and the associated biomarkers are ranked according to their weighted loading factors. Finally, motif enrichment analysis is conducted to evaluate the extracted biomarker candidates in terms of the enrichment of disease-related transcription factors.

### Independent component analysis (ICA)

Consider a gene expression data matrix **X **= [*x*_ji_], whose rows correspond to different microarray samples, and columns correspond to individual genes. ICA decomposition model can be mathematically formulated as (assuming noiselessness for simplicity):

(1) **X**_*N *× *L *_= *A*_*N *× *M*_**S**_*M *× *L*_,

(2) **U**_*M *× *L *_= *W*_*M *× *N*_**X**_*N *× *L*_,

where Equation (1) describes the linear combination model with mixing matrix ***A***, and Equation (2) the decomposition model with de-mixing matrix ***W***. ***S***, **X **and **U **are independent components, mixtures, and estimated independent components, respectively. *M *is the number of independent components, *N *the number of samples and *L *the number of genes.

In microarray data analysis, an ICA model could be interpreted as the expression value of an individual gene *i *under condition *j *(**x**_*i*_(*j*)) is the summation of different linear modes in ***A ***at condition *j *(**a**_*k*_(*j*)) weighted by independent loading factors *s*_*ik *_in ***S***[8], as shown below:

(3) $$\begin{array}{cc} x_{i}(j)=\sum_{k=1}^{M}s_{ik}a_{k}(j), & i=1,...,L;j=1,...,N. \end{array}$$

The linear modes in ***A ***might reflect distinct regulatory mechanisms involved in gene regulation, such as transcription factor (TF) activities. The FastICA algorithm [21] can be utilized to obtain ***A ***and ***S ***based on the assumption that the components are statistically independent and have non-normal distributions (typically super-Gaussian). This assumption is biologically plausible as most genes are not expected to change dramatically. Only the genes involved in distinct regulatory mechanisms will change, producing super-Gaussian distributions in microarray data.

Several methods have been developed to associate a set of genes with a specific linear mode [10,12,22]. These methods each assume that genes with the highest absolute loading values are the significant genes associated with linear mode **a**_*k*_. In this paper, genes are ranked by a modified criterion based on the same assumption as described in the next subsection.

### Knowledge-guided multi-scale ICA

Since ICA is an unsupervised method, it is difficult to determine which linear modes are related to specific biological functions. To identify the biomarkers relevant to a specific biological function, prior knowledge could provide guidance for any computational method. In this approach, we will collect a KGP containing genes strongly associated with the disease and use these to guide the ICA approach for disease-relevant biomarker identification. Notice that the total connection strength of the knowledge genes associated with a disease-relevant linear mode would be larger, in principle, than that of irrelevant linear modes. Based on this observation, the most knowledge-relevant linear mode can be determined from the estimated ICA modes and the associated genes can then be extracted.

However, if we apply ICA to the entire molecular profile, the estimated linear modes will likely reflect the joint effect of several biological functions, even for the most knowledge-relevant mode, because many disease-irrelevant but differentially expressed genes co-exist in the data. Conversely, biomarkers should be involved in several different linear modes in relation to underlying biological processes. Therefore, it is reasonable to first separate the entire profile into sub-populations. We can then find the specific ICA linear modes from different subsets of genes rather than from the whole gene population; this approach is referred to as the "multi-scale ICA" approach in this paper. Since these modes will be associated with different parts of the knowledge genes in the KGP, they are more suitable for biomarker identification. Clustering methods, such as k-means clusterin and SOMs, can be used to form the subsets of genes, with the assumption that the genes involved in similar biological functions are more likely to exhibit similar expression patterns than genes involved in different biological functions.

Our method can be mathematically described as follows. Assume a whole gene population ***G ***in a microarray data **X **has been clustered into *n *subsets, ***G***_1_, ***G***_2_, ..., ***G***_*n*_. For each subset ***G***_*i *_(*i *= 1, ..., *n*), we apply ICA to find the most knowledge-relevant linear mode **a**_*j *_according to the total connection strength of the knowledge genes in this subset. Thus, the index *j *can be obtained by

(4) $$\begin{array}{cc} j=\underset{m}{\arg\max}(\sum_{g\in K_{i}}\left|s_{gm}\right|) & m=1,...,M_{i}, \end{array}$$

where *s*_*gm *_is the loading factor for gene *g *associated with linear mode **a**_*m*_, *K*_*i *_the subset of knowledge genes in the *i*^*th *^cluster, and *M*_*i *_the number of independent components in the *i*^*th *^cluster.

Then each gene *g *in this subset is assigned a score *c*, which is defined as follows:

(5) $$\begin{array}{ccc} c_{g}=w_{i}*\left|s_{gj}\right|, & g\in G_{i}, & w_{i}=\frac{\left|K_{i}\right|}{\left|K\right|}, \end{array}$$

where *w*_*i *_is a weight to represent the significance of the linear mode in the *i*^*th *^subset associated with the prior knowledge. Here we define *w*_*i *_as the proportion of all knowledge genes in this subset with respect to the entire KGP (*K*). Once the knowledge-relevant linear modes in all subsets are determined, each gene will have a score assigned and we rank the genes in terms of their scores. The larger the score, the more strongly the gene is related to the biological process.

A key issue in this method is how to determine the optimal cluster number when forming the subsets of genes. In this paper, we determine the optimal cluster number by a cross-validation approach. Specifically, we assume the optimal cluster number is in some range, from 1 to an upper limit. For each cluster number, the knowledge genes are randomly stratified into a training gene set (as our partial prior knowledge gene set) and a test gene set by a ten-fold cross-validation approach. The method is applied with the partial prior knowledge genes to rank the whole gene population, and prediction accuracy is tested on the test gene set. The above procedure is repeated 10 times, once for each left out fold, and an average accuracy over the ten folds is reported. We select the number with the highest average accuracy as the optimal cluster number for clustering. The upper limit of cluster numbers should be cautiously determined by the number of knowledge genes and the number of genes in the full profile. If the number of clusters is too large, it will lose the ability to infer novel biomarkers. An extreme case is that each individual gene forms a cluster and then we can only obtain the correct ranks for known genes. Genes not in the KGP will be randomly ranked, which is not informative at all for biomarker identification. If the cluster number is too small, the estimated linear modes may be incorrect due to the presence of many irrelevant genes. In our experiments, we set the upper limit as 10 for the yeast cell cycle data set and 15 for the ovarian cancer microarray data set, respectively.

### Knowledge gene pool (KGP)

Each KGP is a collection of those genes that are potentially most strongly related to a specific disease. Usually there are thousands of genes in microarray data and most of them are not relevant to a specific disease even though they exhibit changes in gene expression level. The knowledge gene pool is an important asset for data analysis since it helps reduce many false positives. However, in most cases, little prior knowledge can be obtained, and the available knowledge is usually neither complete nor sufficiently accurate to fully define the specific disease under study. Thus, the KGP is best used as a guide for biomarker identification. In our studies, the KGP is primarily constructed from the published biological literature or from databases such as Ingenuity Pathway Analysis (IPA; Ingenuity Systems: ) and the TRANSFAC 11.1 Professional Database [23].

### Evaluation by motif enrichment analysis

For microarray data analysis, there is often no ground truth (i.e., true biomarkers known to be related to a specific biological process or disease under study) available for us to evaluate the performance of a biomarker identification method. However, we know that gene expression is often regulated by transcription factors (TFs), proteins that bind to promoter or enhancer sequence elements upstream of genes and either activate or inhibit gene expression. In this paper, with the motif information provided, we have designed a statistical test to evaluate the enrichment of transcription factors for a gene set identified. A gene-transcription factor matrix ***M ***is generated where each element in the matrix, *m*_*gf*_, represents how well the upstream sequence of a gene *g *matches the motif that a transcription factor *f *binds to. For human genes, 2 Kbp upstream regions from the transcription start sites (TSSs) of the genes are extracted from the UCSC genome databases [24]. Match™ [25] is then used to search the transcription factor binding site (TFBS) by its position-weighted matrices (PWMs) in a gene's upstream region, which outputs the scores of core similarity and matrix similarity for each matched motif. Since one TF may have multiple TFBSs, we use the summation of average scores of core similarity and matrix similarity to set the final value of *m*_*gf*_.

Given a gene set *S *extracted by a computational method, a statistic to measure the enrichment of a specific transcription factor *f *is defined as

(6) $$e_{f}=\sum_{g\in S}m_{gf}.$$

To calculate the statistical significance (p-value), we need to form a null distribution. The null hypothesis is that the gene set is randomly generated from the gene population and there is no significant enrichment of the transcription factor *f*. We randomly select gene sets with same size of *S *from the baseline gene population, and repeat *B *times to generate the corresponding null statistic enrichment score $e_{f}^{0b}$, for *b *= 1, ..., *B*. The null hypothesis distribution is assumed to be symmetric in this study. The p-value can be obtained for each gene set by calculating the probability that a null gene set has a statistic more extreme than the observed statistic. Mathematically, the p-value can be calculated by:

(7) $$p_{S}=\frac{\text{number of members in }\{b:e_{f}^{0b}>e_{f},b=1,...,B\}}{B}.$$

### Baseline experiments and evaluation method

To evaluate the performance of our proposed approach, EDGE algorithm [3] was first considered as a comparison method since it was specially designed to identify statistically significant genes from time-course microarray data. However this comparison is insufficient due to that EDGE does not incorporate knowledge genes to provide guidance for biomarker identification. On the other hand, given partial prior knowledge genes, traditional supervised classification methods are not suitable to predict whether a gene is related to prior knowledge because there is no true negative gene available. Therefore, we design three baseline biomarker identification methods that incorporate partial prior knowledge for a fair comparison. The first baseline ICA method is designed to evaluate if our multi-scale strategy by clustering offers an improved performance for biomarker identification. Two correlation methods with or without clustering are then implemented to identify the genes exhibiting similar patterns with partial prior genes, compared to the ICA approach focusing on regulatory mode identification. Specifically, the first method is a baseline ICA method where ICA is applied to the entire expression profile and the partial prior knowledge is used to find the most knowledge-relevant linear mode by Equation (4). Genes are ranked according to their absolute connection strengths associated with this linear mode. The second method estimates the correlation with the partial prior knowledge genes without clustering (baseline correlation method-1). Genes are then ranked based on their absolute correlation coefficients between an individual gene expression profile and the average profile of partial prior knowledge genes. However, taking the average profile of all knowledge genes may reduce the sensitivity of detection, especially when the genes in KGP are not similar to each other. To overcome this problem, the third baseline method is a weighted correlation method based on a clustering approach (baseline correlation method-2). Similar to the multi-scale ICA method, the entire gene population is grouped into several sub-populations and a gene in each cluster is assigned a score. The score is the weighted absolute correlation coefficient between an individual gene expression profile and the average profile of partial prior knowledge genes in this cluster. The weight is then calculated using Equation (5) and genes are ranked according to their scores.

Given a ranked gene list and knowledge gene set, we can use the Receiver Operating Characteristic (ROC) curve [26] and the area under the curve (AUC) to measure the test accuracy for each biomarker identification method. ROC curve is a graphical plot of true positive rate (TPR) vs. false positive rate (FPR). AUC is an important performance measure that provides an overall measure of accuracy for the test. Given a ranked gene list (*g*_1_, *g*_2_, ..., *g*_*n*_) with a total of *n *genes and the ground truth gene set G_*k *_with *k *genes, true positive rate and false positive rate, when selecting top *i *genes *G*_*i *_in the list, are calculated as follows:

(8) $$TPR(i)=\frac{\left|G_{i}\cap G_{k}\right|}{k},$$

(9) $$FPR(i)=\frac{i-\left|G_{i}\cap G_{k}\right|}{n-k}.$$

## Results and discussion

We applied our knowledge-guided multi-scale ICA method to two gene expression profiling studies: (1) a yeast cell cycle microarray data set [27] and (2) an Rsf-1-induced microarray data set. The yeast cell cycle data set consists of the expression of 6178 Open Reading Frames (ORFs) during the cell replication cycle in the budding yeast (Saccharomyces cerevisiae). The data set consists of 77 samples corresponding to various experiment conditions. Approximately 800 genes have been identified as cycle-regulated genes; among these 104 genes have been well studied [27]. We us The goal of this experiment is to identify the cell cycle-regulated linear modes and then extract the corresponding genes associated with the cell cycle. We used the 104 genes as our training knowledge gene set and the remaining 704 genes as an independent test set for evaluation.

The Rsf-1-induced microarray data set was acquired and analyzed in our experiment. The dataset was generated using Affymetrix Human Genome U133 Plus 2.0 Arrays from an expression profiling study of ovarian cancer at the Johns Hopkins Medical Institutions. The study was designed to identify Rsf-1 regulated genes in ovarian cancer; Rsf-1 (also known as HBXAP) is a newly discovered gene frequently amplified in ovarian cancer [28]; the protein participates in chromatin remodeling which is essential for a variety of cellular functions including transcription, DNA replication, and DNA repair. The data set is composed of 7 samples with two biological conditions (Rsf-1-induced and not Rsf-1-induced) and four time points at 0 hour, 6 hours, 18 hours, and 30 hours. We used Affymetrix's Probe Logarithmic Intensity Error (PLIER) algorithm with quantile normalization to preprocess the original intensity data for gene expression measurements [29]. After the preprocessing, we obtained expression measurements of 54,675 probe sets for each sample.

The EDGE algorithm was first applied to select statistically significant expressed genes from yeast cell cycle data and Rsf-1 induced ovarian cancer data, respectively. After ranking all genes in terms of their q-values estimated from EDGE, we calculated AUC values for yeast cell cycle-related genes and ovarian cancer-related genes, respectively (see below). As a result, both AUC values are relatively low (around 0.5), which indicates that the genes identified from pure data-driven methods (such as EDGE; without prior knowledge guidance) may not show strong biological relevance.

### Yeast cell cycle data

**Figure 2:**
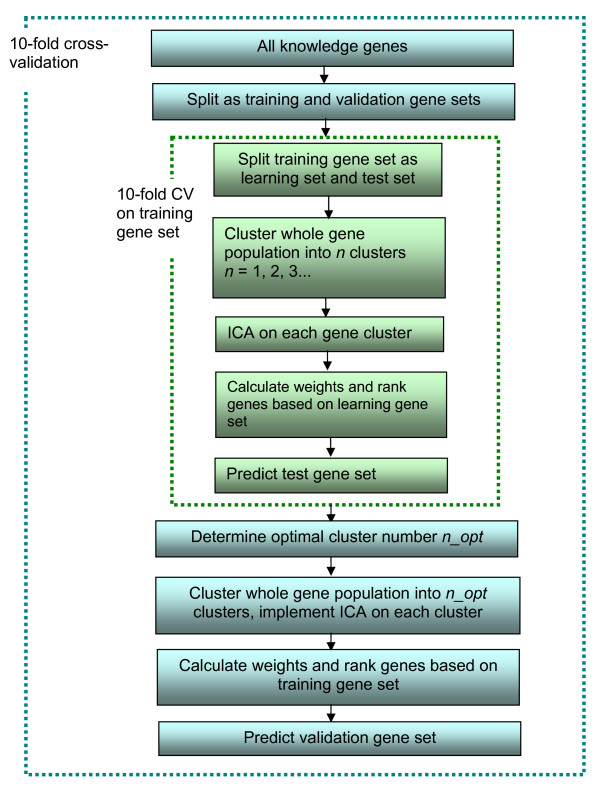
**Procedure of ten-fold cross-validation.** The optimal number of clusters is determined by a nested ten-fold cross-validation on training gene set.

To reduce computational complexity, k-means clustering was used to form the subsets of genes for both datasets. The number of independent components in the FastICA algorithm was set to five for this dataset, since our previous dimension estimation approach with a stability analysis procedure [16] showed that five independent components are sufficient to describe the gene expression data. We first conducted ten-fold cross-validation on the well studied 104 cell cycle-related genes. For each fold, the optimal cluster number is determined by a nested cross-validation procedure on the training gene set, as illustrated in Fig. 2. The number of clusters ranges from 1 to 10. Notice that when the number is 1, no clustering is needed and the algorithm reduces to the baseline ICA method. Each ten-fold cross-validation is repeated 10-times with different randomly chosen stratified sets of knowledge genes. Since the k-means clustering method generates different results depending on its random initialization, we repeat the procedure ten times with different initializations to obtain more reliable results. The results reported here are the average results of the ten different initializations.

**Figure 3:**
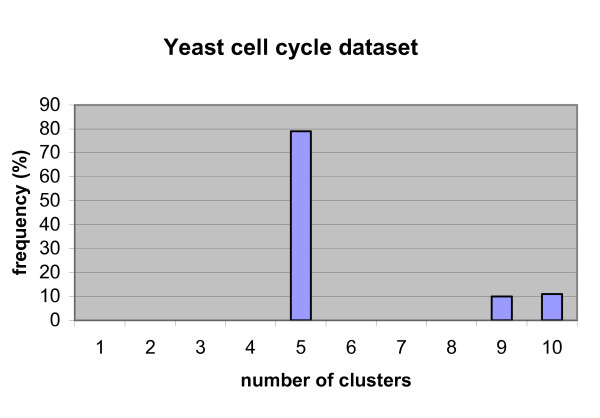
Histogram of determined optimal number of clusters in ten-fold cross- validation on yeast cell cycle data set.

**Figure 4:**
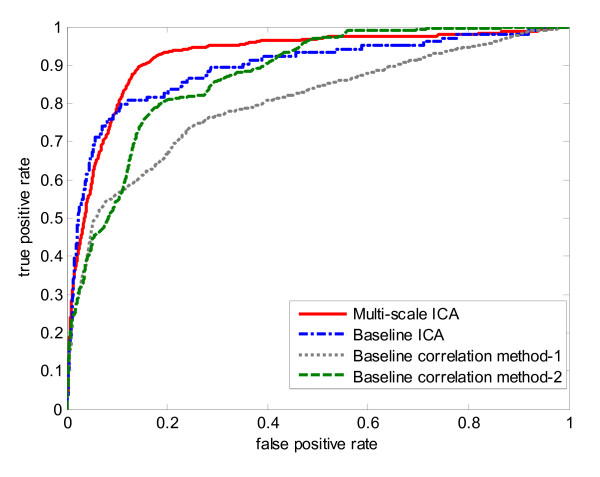
**ROC curves of ten-fold cross-validation for four biomarker identification methods on training knowledge gene set of yeast cell cycle data set.** Solid line represents the multi-scale ICA method; dash-dotted line represents the baseline ICA method; dotted line represents the correlation method-1; dash line represents the correlation method-2.

The resulting average AUC value of ten-fold cross-validation on 104 genes is 0.9206 with standard deviation of 0.0470. Fig. 3 shows the histogram of determined optimal number of clusters during the ten-fold cross-validation procedure. From the figure we can see the most frequent number of clusters is five. Then we implemented three baseline methods for ten-fold cross-validation as comparisons. For baseline correlation method-2, we chose the optimal cluster number from the multi-scale ICA method for a fair comparison. The ROC curves of ten-fold cross validation for the two baseline correlation methods, the baseline ICA method, and our multi-scale ICA method are shown in Fig. 4. The ROC curves show that the multi-scale ICA method outperforms the baseline correlation method-2, and that the baseline ICA approach is better than the baseline correlation method-1. Overall, the proposed multi-scale ICA method significantly outperforms all three baseline methods as estimated by the Kolmogorov-Smirnov (K-S) one-sided test (Table 1).

**Table 1:** P-values of Kolmogorov-Smirnov test for different methods on yeast cell cycle data using ten-fold cross-validation

Method 1	Method 2	P-values of K-S test
Optimal ICA	Baseline ICA	< 1e-10
Optimal ICA	Correlation method 1	< 1e-10
Optimal ICA	Correlation method 2	< 1e-5

To further test the generalizability of our method, we conducted ten-fold cross-validation on the 104 genes using a subset of samples. The original data set includes 77 samples synchronized by three independent methods: *α *factor arrest, elutriation and arrest of a *cdc *15 temperature-sensitive mutant [27]. We selected 63 samples from all the samples by excluding those samples under elutriation condition. The resulting average AUC value is 0.9157 with standard deviation of 0.0458. Also the most frequent optimal cluster number is five (with a frequency of 65%), which shows a great consistency when compared to the result using all the samples.

**Figure 5:**
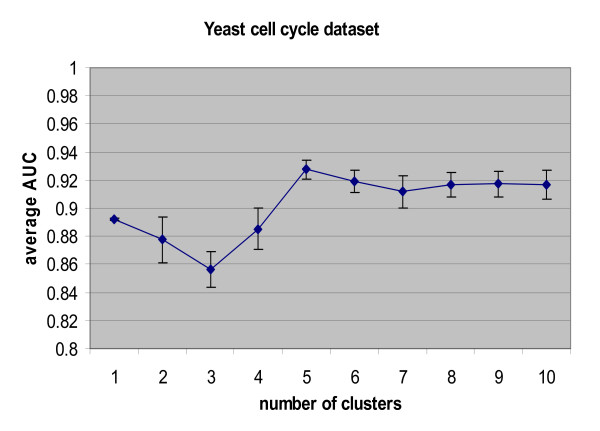
**Average area under the curve (AUC) values using ten-fold cross-validation with different numbers of clusters on 104 knowledge genes.** The knowledge-guided multi-scale ICA method is applied to yeast cell cycle data set for the identification of cell cycle-related genes.

**Figure 6:**
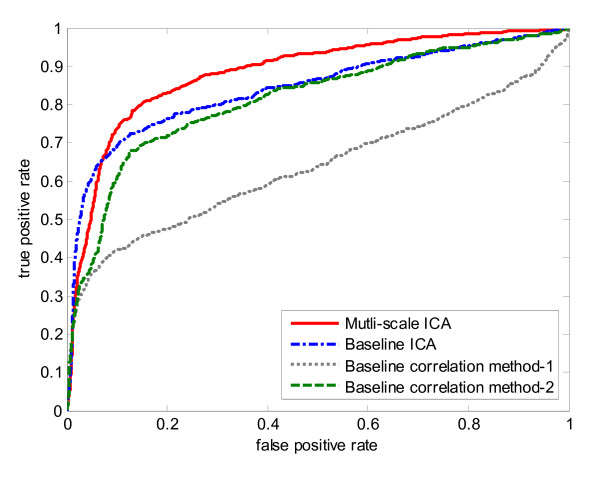
ROC curves of four biomarker identification methods on yeast cell cycle data set with an independent test gene set.

All 104 knowledge genes were then used as a training set in the algorithm to test 704 cell cycle-related genes for all four methods. During the training, we still used tenfold cross-validation to determine the optimal number of clusters. Fig. 5 shows the average AUC values and their standard deviations in ten-fold cross-validation across different number of clusters. From the figure we can see that the average AUC (standard deviation), starting at 0.892 (0.0006) for the full gene population, decreases a little at two and three clusters. The AUC increases gradually and reaches the peak of 0.9274 (0.0071) at five clusters, at which it remains constant. So the optimal number of clusters for multi-scale ICA approach is five. Then an independent evaluation was performed on the test gene set and the ROC curves for these four methods was calculated when the cluster number is five (Fig. 6). The ICA-based methods significantly outperform the baseline correlation methods, and the multi-scale ICA is the best method when compared with the three baseline methods (Table 2).

**Table 2:** P-values of kolmogorov-Smirnov test for different methods on yeast cell cycle data using an independent test gene set

Method 1	Method 2	P-value of K-S test
Optimal ICA	Baseline ICA	< 1e-10
Optimal ICA	Correlation method 1	< 1e-10
Optimal ICA	Correlation method 2	< 1e-10

**Figure 7:**
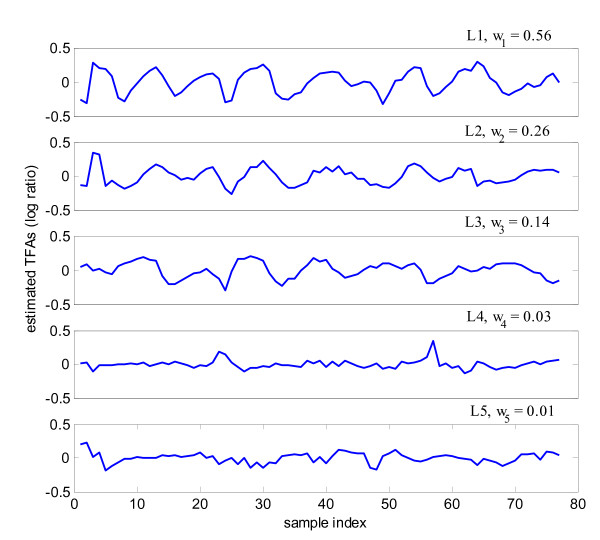
**Five cell cycle-related linear modes in the proposed multi-scale ICA approach on yeast cell cycle data set.** The weight is also listed in the figure for each linear mode.

We examined in detail the extracted knowledge-relevant linear modes and the biological functions of their associated cell cycle-regulated genes. Fig. 7 shows five knowledge-relevant linear modes and their weights as identified when the number of clusters is set at the optimum number of five (Fig. 5). The top three linear modes have much higher weights than the lower two modes and their estimated TFAs clearly show periodic patterns related to cell cycle. We examined the biological functions of these well-known cell cycle-regulated genes associated with these three linear modes. The majorities of genes in linear mode L3 are associated with the M/G1 boundary or are known transcriptional targets of STE12/MCM1. Most of the genes in linear mode L1 are SCB/MCB regulated in late G1 and S phase. Finally, many genes in linear mode L2 are in S/G2 and G2/M phases. In summary, we can see that the linear modes L3, L1, and L2 correspond to different biological functions in cell cycle process.

The top 10 genes selected by multi-scale ICA method are listed in Table 3. Among them, four genes (CLN2, MCD1, POL30 and RNR1) are in the known training gene set. All other genes (CSI2, PRY2, YOX1, TOS4, AXL2 and CRH1) are the genes related to cell cycle beyond our training gene set, i.e., in the test gene set. The results show that our method is effective at finding novel biomarkers beyond knowledge, which is clearly an important feature of the proposed approach for novel biomarker identification beyond prior knowledge. In most of cases, especially for human disease, knowledge genes are limited and we need to infer the new ones from partial knowledge for biomarker discovery.

**Table 3:** Top10 genes selected by the proposed multi-scale ICA method on yeast cell cycle data

Rank	ORF	Name	Short Description
1	YPL256C	CLN2	CycLiN; G1 cyclin involved in regulation of the cell cycle
2	YOL007C	CSI2	Chitin Synthesis Involved; protein of unknown function
3	YKR013W	PRY2	Pathogen Related in Yeast; protein of unknown function
4	YDL003W	MCD1	Mitotic Chromosome Determinant; expression is cell cycle regulated and peaks in S phase
5	YML027W	YOX1	Homeodomain-containing transcriptional repressor
6	YBR088C	POL30	POLymerase; proliferating cell nuclear antigen (PCNA)
7	YLR183C	TOS4	Target of SBF; promoters of some genes involved in pheromone response and cell cycle;
8	YIL140W	AXL2	AXiaL budding pattern; glycosylated by Pmt4p; potential Cdc28p substrate
9	YGR189C	CRH1	Congo Red Hypersensitive; cell wall protein; putative chitin transglycosidase
10	YER070W	RNR1	RiboNucleotide Reductase; the RNR complex catalyzes the rate-limiting step in dNTP synthesis and is regulated by DNA replication and DNA damage checkpoint pathways via localization of the small subunits

### Rsf-1-induced gene expression data

#### Knowledge gene pool (KGP)

To construct the KGP, we started with the known gene Rsf-1 and its related genes, NF-kappa B (NFKB1) and SMARCA5 (also known as hSNF2H) as reported in [30], to search the databases. We used Ingenuity Pathway Analysis (IPA) to extract 95 genes that are thought to be directly related to NFKB1 and SMARCA5. Note that there is no network related to Rsf-1 in the current IPA database. We also included 43 genes from TRANSFAC 11.1 Professional Database [23], whose protein products are transcription factors biologically relevant to ovarian cancer as reported in literature. Hence, our KGP consists of 141 distinct Affymetrix probe set identifiers that represent the expression values for the 138 genes.

#### Multi-scale ICA results

**Figure 8:**
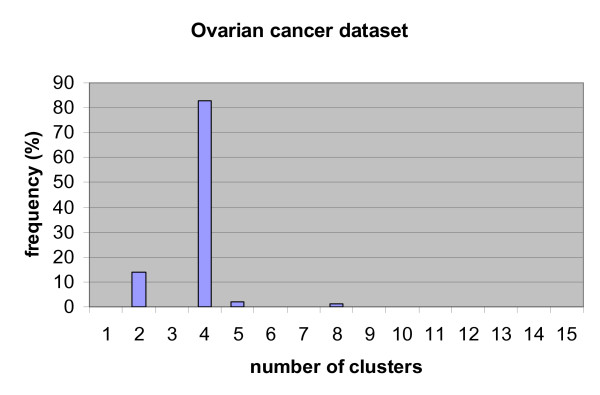
Histogram of determined optimal number of clusters in ten-fold cross- validation on ovarian cancer data set.

**Figure 9:**
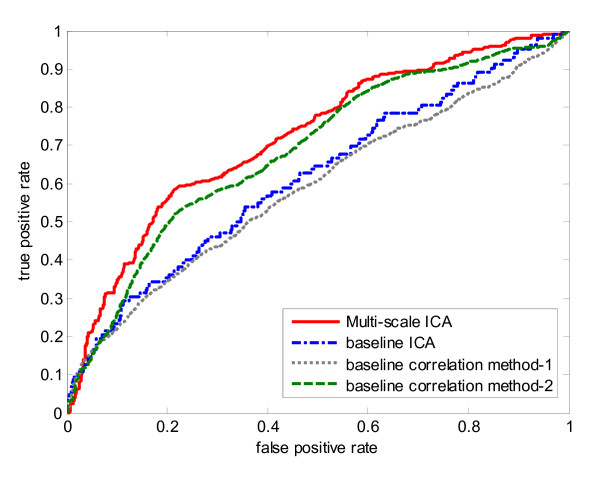
**ROC curves of ten-fold cross-validation for four biomarker identification methods on knowledge gene set of ovarian cancer data set.** Solid line represents the multi-scale ICA method; dash-dotted line represents the baseline ICA method; dotted line represents the correlation method-1; dash line represents the correlation method-2.

We used 'tanh' nonlinearity in the FastICA algorithm: other parameters were set at their default values. The number of the independent components is set to a maximum value of 6 due to the limitation of sample size. Ten-fold cross-validation was conducted on our partial prior knowledge genes, where the optimal cluster number was determined by a nested cross-validation approached for each fold as shown in Fig. 2. The number of clusters was set from 1 to 15. We also repeated 10 times for ten-fold cross-validation and k-means clustering in order to generate more reliable results. The resulting average AUC is 0.7203 with standard deviation of 0.0804. Fig. 8 shows the histogram of determined optimal cluster number in the ten-fold cross-validation procedure and we can see that the most frequent cluster number is 4. We compared the ROC curves for the two baseline correlation methods, the baseline ICA and the multi-scale ICA for ten-fold cross-validation (Fig. 9). The results in Table 4 show that multi-scale ICA method performs significantly better than baseline ICA method and baseline correlation method-1 with p-value < 1e-10, while performing marginally better than baseline correlation method-2 (p-value = 0.0037). Since baseline correlation method-2 also calculates clustered average profiles of the prior knowledge genes, this result indicates that the multi-scale approach by clustering is an effective strategy to improve the performance for ovarian cancer-related biomarker identification. On the other hand, a major weakness in baseline correlation method-1 lies in that the average profile of all prior knowledge is used when their expression profiles are not similar to each other.

**Table 4:** P-values of Kolmogorov-Smirnov test for different methods on Rsf-1-induced ovarian cancer microarray data

Method 1	Method 2	p-value of the K-S test
Optimal ICA	Baseline ICA	< 1e-10
Optimal ICA	Correlation method 1	< 1e-10
Optimal ICA	Correlation method 2	0.0037

#### Evaluation by motif analysis

**Figure 10:**
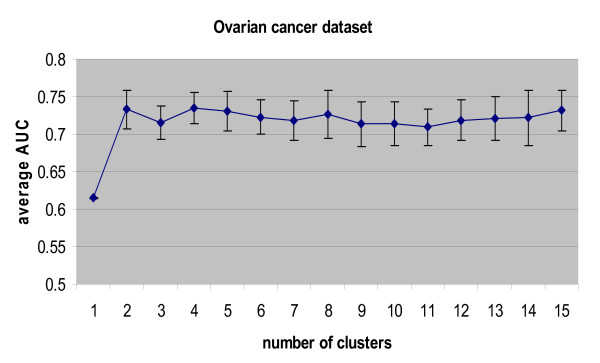
**Average AUC values using ten-fold cross-validation across different numbers of clusters.** The knowledge-guided multi-scale ICA method is applied to Rsf-1-induced ovarian cancer microarray data set for the identification of disease-specific biomarkers.

**Figure 11:**
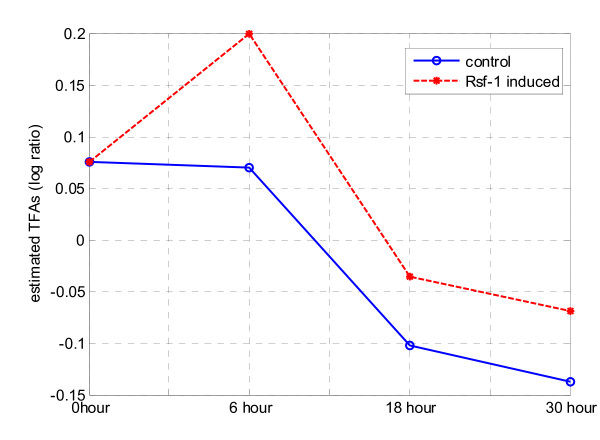
**Estimated knowledge-related TFAs using baseline ICA method.** X-axis represents the time and Y-axis represents the estimated TFAs.

**Figure 12:**
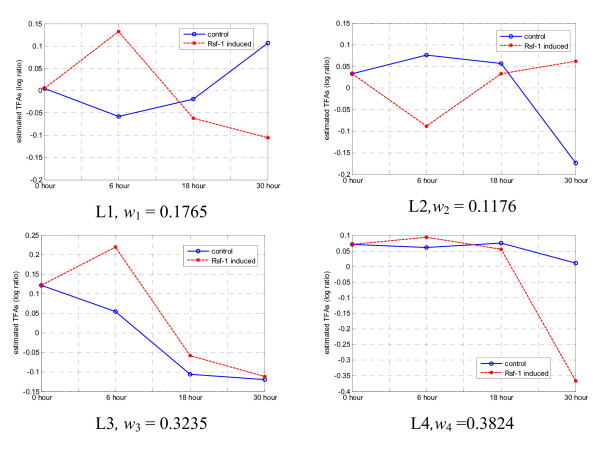
**Estimated four knowledge-related TFAs using the proposed muti-scale ICA method.** X-axis represents the time and Y-axis represents the estimated TFAs.

All knowledge genes were used as the training set in the algorithm to rank the whole gene population for all four methods. During the training, we still used ten-fold cross-validation to determine the optimal number of clusters in multi-scale ICA method. Fig. 10 shows the average AUC values and their standard deviations obtained with different numbers of clusters for the ten-fold cross-validation; the average AUC (standard deviation), starting at 0.6146 (0.0004) for the whole gene population, increases to 0.7329 (0.0253) at two clusters and reaches the maximum value of 0.7343 (0.0210) at four clusters, and remains almost constant thereafter. Therefore, the optimal number of cluster for the multi-scale ICA approach was selected as four. Specifically, we examined estimated linear modes from ICA methods. Fig. 11 shows the estimated knowledge-related TFAs using baseline ICA method and Fig. 12 shows the estimated four knowledge-related TFAs and their weights using our multi-scale ICA method. We observe that one of the TFA patterns in Fig. 12 (L3) is similar with that in Fig. 11, which indicates that multi-scale ICA method can estimate more TFAs for knowledge-related genes than baseline ICA method. Four different linear modes and their weights in Fig. 12 also indicate that the expression patterns of the genes in KGP are not similar to each other, which seems to be the major reason behind that baseline correlation method-1 (using the average profile of all prior knowledge) underperforms other methods.

**Figure 13:**
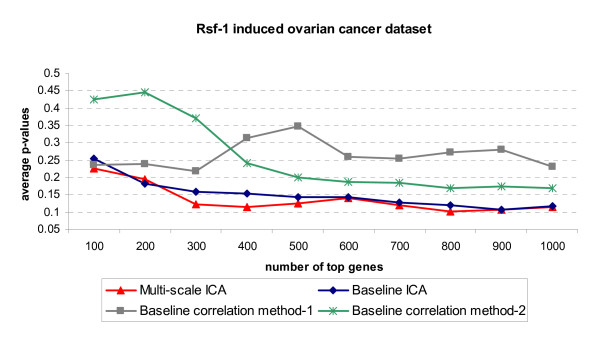
Average p-value of TF enrichment for different gene sets associated with different methods on Rsf-1-induced ovarian cancer microarray data set.

For the final ranked gene lists, we performed motif enrichment analysis to evaluate the performance of each of the four different methods for biomarker identification. Specifically, among 43 ovarian cancer-related TFs extracted from TRANSFAC 11.1 Professional Database [23], 14 TFs have their PWMs available and we generated the gene-TF matrix ***M ***for them. For each TF, a PWM was chosen from the vertebrate non-redundant profiles. Table 5 lists their TRANSFAC PWM entry IDs and the corresponding TF descriptions. To increase the statistical power, we conducted multiple tests by selecting different gene sets with different sizes for different gene selection methods. The number of genes in each gene set ranges from 100 to 1,000 and the average p-values for 14 TFs are reported. Fig. 13 shows the average p-values of TFs enrichment for different gene sets selected by different methods. Both ICA methods outperform the baseline correlation methods in terms of finding more enriched ovarian cancer-related TFs binding sites. Moreover, our multi-scale ICA method is slightly better than baseline ICA method for motif enrichment. It is worth noting that although both multi-scale ICA and baseline ICA methods can extract ovarian cancer-related biomarkers with significant motif enrichment, multi-scale ICA method can help reveal more biomarkers related to ovarian cancer. For this experiment, it is also expected to have similar TF enrichment from both methods, since one common linear mode is revealed by both methods (i.e., the mode in Fig. 11 is very similar with the L3 mode in Fig. 12). From the pattern of this common mode, we postulate that this is a major mode related to *RSF-1*-induced ovarian cancer. Therefore, the genes extracted from this mode will show a similar significance level in TF enrichment (as shown in Fig. 13). However, the multi-level ICA approach can extract other linear modes related to ovarian cancer (see Fig. 12). Apparently, the biomarkers related to these other modes cannot be identified with the baseline ICA approach. This can be supported by the ROC curves in Fig. 9, showing an improved performance of using multi-scale ICA approach compared to that of using baseline ICA approach.

**Table 5:** Ovarian cancer-related TFs and their TRANSFAC entry IDs & descriptions

Index	TF Name	PWM Access No.	Consensus Binding Site	Factor Description
1	AP-2	M00189	MKCCCSCNGGCG	Activator protein 2
2	AP-2alpha	M00469	GCCNNNRGS	Activating enhancer binding protein 2 alpha
3	AP-2alphaA	M01045	ANNGCCTNAGGSNNT	Activating protein 2, AP-2A, Ker-1
4	AP-2gamma	M00470	GCCYNNGGS	Activator protein 2gamma, ERF-1
5	AP-2rep	M00933	CCCCGCCCCN	Specificity protein1, stimulating protein 1
6	BRCA1	M01082	KTNNGTTG	Breast cancer type 1 susceptibility protein
7	E2F	M00516	TTTSGCGCGMNR	EIIF protein, activator of myc, important for p107 promoter activity
8	Elk-1	M00007	NAAACMGGAAGTNCVH	Elk1, member of ETS oncogene family
9	NF-kappaB	M00774	NNNNKGGRAANTCCCN	Nuclear factor kappa B, p50
10	Sp1	M00933	CCCCGCCCCN	Specificity protein1, stimulating protein 1
11	TGIF	M00418	AGCTGTCANNA	5'-TG-3' interacting factor, TG-interacting factor, TGFB-induced factor
12	c-Rel	M00053	SGGRNTTTCC	Nuclear factor kappa B c-Rel, p68
13	P53	M00272	NGRCWTGYCY	Tumor protein p53, TRP53
14	ER	M00191	NNARGNCANNNTGACCYNN	Estrogen receptor

#### Discussion with biological interpretation

**Figure 14:**
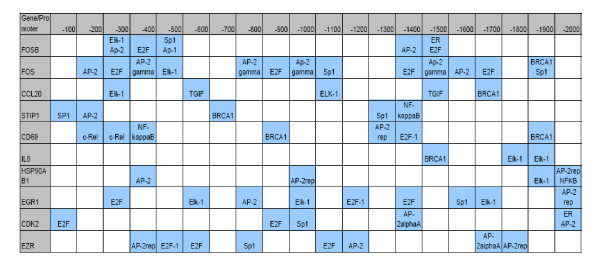
**TFs and their locations in 2 Kbp promoter region for top 10 genes selected by our approach.** The promoter region is represented from -2,000 bp to 0 from TSS and each block in the figure represents a 100 bp region.

**Figure 15:**
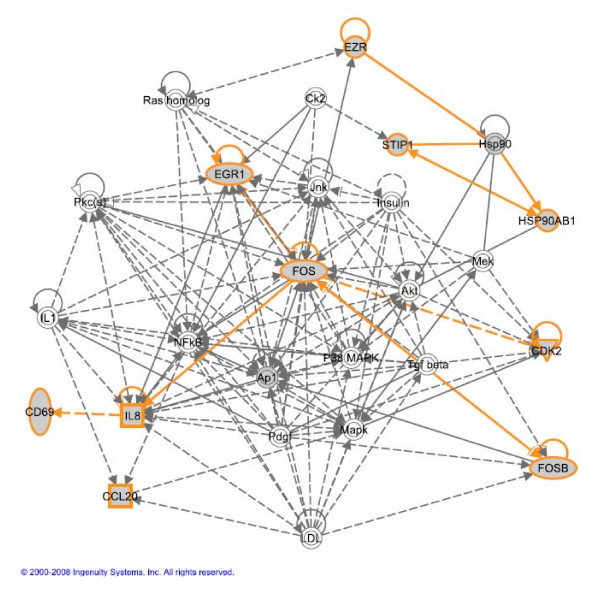
**The network obtained from IPA with all of top 10 genes in Table 6.** Five genes, FOSB, FOS, EGR1, IL8 and CDK2, are highly related to cancer module.

To enable a more detailed analysis, the top 10 genes extracted by optimal multi-scale ICA method are listed in Table 6 and the putative TFs in their promoter regions are shown in Fig. 14. Since none of the genes are in the KGP, they were entered into an Ingenuity Pathways Analysis (IPA) where we found that all of these genes can be incorporated into a single hypothetical network (Fig. 15). The major functions of this network are involved in gene expression, cancer development, and cellular motility. Five genes, FOSB, FOS, EGR1, IL8 and CDK2, are in the cancer module with p-values ranging from 1.84E-7 to 6.5E-3. FOSB and FOS belong to the Fos family that hetero-dimerizes with Jun proteins to form the AP-1 transcription factor complex [31]. AP-1 transcription factors control rapid responses of mammalian cells to stimuli that are associated with proliferation, differentiation and transformation [32]. IL-8 is a member of the C-X-C family of chemokines, and overexpression of IL-8 is observed in subsets of human ovarian cancer cells [33]. Previous studies have shown that the expression of interleukin-8 (IL-8) is directly correlated with the progression of human ovarian carcinomas implanted into the peritoneal cavity of nude mice [34]. The early growth response 1 (EGR1) is a transcription factor that acts as both tumor suppressor and tumor promoter depending on the cellular context. In the experiments of multiple pituitary and ovarian defects in Krox-24 (NGFI-A, Egr-1)-targeted mice, EGR1 was implicated as a novel key regulator of anterior pituitary physiology and that it may play important roles in specific cell lineages [35]. CDK2 is known to be involved in cell cycle regulation and the overexpression of CDK2 is associated with malignancy in ovarian tumors [36].

**Table 6:** Top 10 genes selected by the proposed multi-scale ICA on Rsf-1-induced microarray data

Rank	Probe Set ID	Gene Symbol	Gene Full Name
1	202768_at	FOSB	FBJ murine osteosarcoma viral oncogene homolog B
2	209189_at	FOS	v-fos FBJ murine osteosarcoma viral oncogene homolog
3	205476_at	CCL20	chemokine (C-C motif) ligand 20
4	212009_s_at	STIP1	stress-induced-phosphoprotein 1
5	209795_at	CD69	CD69 molecule
6	211506_s_at	IL8	interleukin 8
7	1557910_at	HSP90AB1	heat shock protein 90 kDa alpha (cytosolic), class B member 1
8	227404_s_at	EGR1	Early growth response 1
9	211804_s_at	CDK2	cyclin-dependent kinase 2
10	208621_s_at	VIL2	villin 2

## Conclusion

Biomarker identification is an important goal in many microarray data analyses. We propose a novel method, knowledge-guided multi-scale ICA, to find relevant biomarkers associated with specific biological functions. We aimed to infer knowledge-relevant regulatory signals and then identify corresponding biomarkers through a multi-scale strategy. A knowledge gene pool is constructed from multiple knowledge sources to help identify disease-specific gene clusters. By applying ICA to multi-scale gene clusters, an examination of the revealed regulatory modes can uncover knowledge of the underlying biological regulatory mechanisms. In addition, we have designed a statistical test procedure to measure the transcription factor enrichment of a selected gene set based on motif information. The approach was successfully applied to two gene expression profile data sets to identify biomarkers: yeast cell cycle microarray data and Rsf-1-induced microarray data. The experimental results show that our method can extract apparently biologically meaningful and condition-related biomarkers. The performance of the proposed method significantly outperforms several baseline methods for biomarker identification. More importantly, the proposed method has notable potential to discover novel biomarkers beyond any partial prior knowledge.

## Authors' contributions

LC and JX formulated the problem and developed the theoretical framework of the algorithm. LC and CW carried out the development and implementation of the algorithm. IS and ZZ directed the application of the algorithm to the ovarian cancer data set. EH, RC and YW provided technical and biological support to the project. All authors participated in the writing of the manuscript, and have read and approved the manuscript.
